# Takotsubo or Stress Cardiomyopathy

**DOI:** 10.1155/2012/637672

**Published:** 2012-09-28

**Authors:** J. P. Bounhoure

**Affiliations:** Service de Cardiologie, Centre Hospitalier de Rangueil, Toulouse University, 31000 Toulouse, France

## Abstract

Many case reports have been published of reversible left ventricular dysfunction precipitated by sudden emotional stress. We have evaluated 10 women hospitalized for acute chest pain and dyspnea, mimicking an acute coronary syndrome, after a severe emotional trigger. Those patients, postmenopausal women, presented ST segment alterations on the EKG, minor elevations of cardiac enzymes, and biomarkers levels. At the coronarography there was not coronary thrombosis or severe stenosis, but the ventriculography showed wall motion abnormalities involving the left ventricular apex and midventricle, in the absence of significant obstructive coronary disease. The course was benign without complication, with a full recovery of left ventricular function in some weeks. These observations, like other reports, demonstrate the impact of emotional stress on left ventricular function and the risk of cardiovascular disease. The cause of this cardiomyopathy is still unknown, and several mechanisms have been proposed: catecholamine myocardial damage, microvascular spasm, or neural mediated myocardial stunning.

## 1. Introduction

Broken heart syndrome or transient stress induced cardiomyopathy is characterized by left reversible systolic dysfunction which appears to be triggered by an intense psychologic stress in the absence of myocardial infarction.

The syndrome is also known under several names, including “Ampulla cardiomyopathy, Takotsubo cardiomyopathy, Left Apical Ballooning Syndrome.” First reported by SATO et al. in the Japanese population in 1980, Takotsubo is a pot with a round bottom and a narrow neck used for trapping octopuses in Japan [1]. Today, many cases have been described worldwide, indicating that is extremely unlikely to be a geographically isolated disease. Owing to its clinical and imaging characteristics mimicking an acute coronary syndrome, apical ballooning syndrome is often misdiagnosed. Despite the frequently dramatic clinical presentation, almost all patients recover fully, and the left ventricular function heavily depressed at presentation, improves rapidly in a period of some days to weeks. The purpose of this paper is to present our experience and to review some published reports about this syndrome [2–4].

## 2. Documents and Methodology

Ten previously healthy patients were admitted to the Coronary Care Unit of the Academic Hospital Center of Rangueil in Toulouse or in the General Hospitals of Midi Pyrénées Area.

Their median age was 52 years (range 48–65 years). Those patients, ten postmenopausal women, were hospitalized for a severe chest pain and acute dyspnea in emergency in the Coronary care Unit. All were evaluated by means of serial electrocardiograms and serial measurements of cardiac isoenzymes, including creatine kinase, creatine kinase MB fraction, and cardiac troponins I and T. All these patients underwent in emergency, coronary angiography and left ventriculography. A two-dimensional trans thoracic echocardiography was realized within the 24 hours after the onset of symptoms.

The two oldest women had been treated for hypertension and dyslipidemia, and the eight others had not vascular risks factors. 

The causal emotional stress was an acute emotional distress after their sudden accidental son's death in two cases, an armed robbery for two patients, financial loss for two others patients, car accident for one patient, and dramatic explosion of a factory in Toulouse causing severe acute pain for two patients. Acute dyspnea and pulmonary sub-oedema with severe left ventricular dysfunction were associated to chest pain in six cases. The cardiac markers were slightly elevated suggestive of mild cardiac injury with a mean peak of troponins of 1,20 ng/mL. The peak creatine kinase level was 150 mL/L/(range from 104 to 283), and the mean peak cKMB level was 8 ng/mL.

At the coronarography, seven women had absolute normal coronary arteries, three mild luminal irregularities in the proximal left anterior descending artery. No patient had angiographic evidence of coronary spasm or thrombosis. The contrast left ventriculography revealed apical and mild ventricular akinesis, apical dilatation, with normal contractility of the basis of the heart. Mean ventricular ejection fraction value was 0,35 (range 0,25–0,45). Initial echocardiogram showed a similar contractile pattern with preserved basal function and apical dyskinesia. For only 3 patients it was possible, on hospital days 2 and 3, to measure plasma level of catecholamines: mean value of epinephrine, 1340 pg/mL and norepinephrine, 1570 pg/mL.

 During the outpatients followup, the left ventricular ejection fraction had completely recuperated, and all segments of left ventricle had normal contractility.

Four patients underwent magnetic resonance imaging confirming left ventricular dysfunction in acute phase. This examination demonstrated the absence of myocardial necrosis or late gadolinium-enhanced image.

## 3. Evolution

The prognosis was favourable for all these women, without major complication during the hospital stay and without mortality. All the patients had a good evolution with a treatment by furosemide, betablockers, heparin, and ACE inhibitors. Dyspnea and the signs of pulmonary congestion disappeared after the diuretic treatment, conversely the T wave inversion on EKG resolved very slowly, in mean after 3 months.

 By a median of 6 days after presentation in the hospital, the left ventricular ejection fraction had improved to 0,50. A full recovery of left ventricular function was recuperated to echocardiography within 4 to 6 weeks from the onset of symptoms. None recurrence or other complication were observed during the two years of followup.

## 4. Comments

After the introduction of the term Takotsubo and the description of this syndrome in Japan many cases were reported worldwide. The characteristic feature of this syndrome is the peculiar and transient end-systolic ventricular shape that inspired the name. The prevalence of this disease is unknown. In Japan it is estimated to be as high as 2% of hospital admissions for chest pain and acute ST changes. A strict predilection for female gender and for postmenopausal women is one of the hallmark of Takotsubo cardiomyopathy [3–5].

Postmenopausal women reported to make up over 90% of the cases in most series. The reason for the female predominance remains unknown although a lack of oestrogen in the postmenopausal phase seems to play a pathogenic role. Patients suffering from broken heart syndrome have a clinical presentation very similar to that of an acute coronary syndrome. Most reported patients with this cardiomyopathy were hospitalised after an episode of an important psychological or emotional stress. The acute chest pain or left ventricular dysfunction follows an acute mental trigger: unexpected loss of a close relative, dispute with another person, devastating financial loss, or a physical stress, sepsis, trauma, or cerebrovascular accident. But the lack of preceeding trigger does not exclude the diagnosis of Takotsubo cardiomyopathy. Chest pain is often accompanied by an acute dyspnea, palpitations arrhythmias, collapses and syndrome of heart failure. A true cardiogenic shock requiring circulatory assistance was reported [4, 5].

 The syndrome mimics acute myocardial infarction in the absence of obstructive epicardic coronary atherothrombosis. Sharkley et al. studied the EKG of 59 patients with this diagnosis. On admission 56% had ST elevation, 27% T-wave inversion, 10% new Q waves or abnormal R-wave progression [6]. During the recovery phase, new or deepening T-wave inversion in precordial leads was the most frequent EKG feature. The absence of reciprocal changes in the inferior leads and a ratio of ST segment elevation in leads from V4–V6 to V1–V3 >1 has also been considered highly specific of the Takotsubo syndrome.

 Plasma levels of catecholamines and their metabolites are elevated in the acute phase in 74% of patients when these concentrations are measured. Wittstein et al. compared plasma catecholamines in 13 patients with Takotsubo cardiomyopathy with seven controls hospitalized for acute myocardial infarction with left ventricular dysfunction. They found that catecholamines levels were two or three times higher in patients with left ventricular ballooning syndrome [7]. During the acute phase midventricular left ventricular wall motion abnormalities, apical akinesia or dyskinesia, with preserved or hyperkinetic contractile function of the basal left ventricular segments are characteristic [6–8]. Recent cases reports have described a kind of inverted TK with suppression of basal contraction and hypercontracting left ventricular apex. In these cases, a hypokinetic basis of the heart with an hyperkinetic ventricular apex was reported.

The typical and important finding is the absence of obstructive coronary disease.

 Nevertheless, Ibanez et al. were able to describe the presence of ruptured atherosclerotic plaques in some patients with the use of intravascular ultrasound [9].

Based on their experience a team of the Mayo Clinic proposed some criteria for this diagnosis (Table 1). In the literature, some complications have been described, during the acute phase:

- cardiogenic shock 8%,
- congestive heart failure 8%,
- ventricular tachycardia 3%,
- subite death 3,5%, and
- others rare complications have been reported: left ventricular rupture, apical thrombus formation, distal or cerebral emboli (5-7-10).

## 5. Physiopathology

The pathogenetic mechanisms underlying the development of this transient ventricular dysfunction remain largely unknown. Several theories have been proposed.

- A microvascular dysfunction or a multivessel acute epicardial coronary spasm with transient impaired coronary blood flow has been evocated by some authors [1, 3, 5].
- Elesber et al. have reported an abnormal TIMI myocardial perfusion grade (an angiographic index of myocardial perfusion) in 69% of their patients [8].
- Structural and ultrastructural myocardial changes are rather suggestive of direct catecholamines toxicity, and myocardial sudden stunning.
- Since the first report by Kume et al. about increased catecholamines plasma levels in patients with Takotsubo cardiomyopathy, other studies have confirmed these author's finding reporting increased local release assessed by blood sampling from the aortic root and coronary sinus [10]. The increased susceptibility of the apex to the direct toxic effect of catecholamines is possible with an increased density of beta adrenoreceptors in the apex. Catecholamines may play a role because many patients have an emotional sudden trigger. There is an increasing awareness of a close interaction between cortical brain activity and the heart [11]. Wall motion abnormalities and depressed left ventricular function have been observed in diseases associated with high catecholamines release and plasmatic levels such as a pheochromocytoma and subarachnoid hemorrhage. Elevated catecholamines plasmatic levels decrease the viability of myocytes through cyclic AMP overload. They are also a potential source of oxygen-derived free radicals and in animal models cause myocyte injury, that is attenuated by anti oxidants. The catecholamines have been associated with contraction band necrosis, a form of myocyte injury characterized by hypercontracted sarcomeres, dense eosinophilic transverse bands, and an interstitial mononuclear inflammatory response [10].
- According to Akashi et al., “a number of important questions remain.” “Experimental approaches to mimic the clinical manifestations may provide new insights into the pathogenesis of this syndrome. The development of animal models began to address this issue. The adrenal stimulation given by immobilization in rats mimics the clinical condition of takotsubo cardiomyopathy. Immobilization induces upregulation of immediate early genes, such as c-fos and c-jun in endothelial, myocardial, and coronary muscle cells. The expression of these genes suggests coronary spastic changes and consequent microvascular dysfunction.” [11].
- Akashi et al. have hypothesized that the reduced estrogen levels after menopause explain the predisposition of elderly women to this cardiomyopathy and induced vulnerability to stress. Estrogen supplementation attenuated the immobilization-induced cardiac dysfunction, sympatho adrenal activation, and vagal inhibition.
- “Treatment with estrogen attenuated the immobilization-induced increase in c-fos mRNA expression in the lateral septum, medial amygdaloid nucleus, paraventricular and dorsomedial hypothalamic nucleus, laterodorsal tegmental nucleus, regions that are parts of the central autonomic network and possess immunoreactive estrogen receptors.” These data suggest that reduced estrogen levels play a role in the pathogenesis of this cardiomyopathy [12].
- “But the estrogen hypothesis does not seem sufficient to explain the occurrence—albeit uncommon—of this cardiomyopathy in men.” The hypothesis that catecholamine surge may play an important role in the pathogenesis of the syndrome is supported by many authors.
- The development of transient severe left ventricular outflow obstruction is another mechanism that has been implicated in the pathogenesis of the Takotsubo syndrome. In the setting of massive catecholamine surge, elderly women, who frequently had a sigmoid interventricular septum, could potentially develop severe obstruction, outflow tract leading to apical ischemia as a result of increased wall stress. However, if this was the case, one would expect to document an intraventricular gradient more often than it has been reported in the literature [13]. Nevertheless, this hypothesis could be represented a contributing cause in some cases. Lastly a neural mediated mechanism has been proposed. A pattern of apical dysfunction similar to that of Takotsubo syndrome has been observed in patients with subarachnoid hemorrhage. Interestingly, this has been associated with rupture of aneurysms located in the anterior half of the circle of Willis. These aneurysms overlie the amygdala and the right insular cortex, which control sympathetic outflow to the heart [14]. Although this last hypothesis offers a functional explanation of stress myocardial ischemia and dysfunction, there is not any strong experimental evidence to support it.

## 6. Management

The optimal treatment of broken heart syndrome remains unknown. Initial management should be definitely directed towards the treatment of myocardial ischemia with continuous EKG monitoring, heparin, oral and intravenous nitrates, and betablockers. Cardiogenic shock due to pump failure is treated with standard therapies and in some severe cases, intra-aortic ballon counter pulsation. Anticoagulation should be considered during initial presentation and the acute phase if severe left ventricular dysfunction is present, like the prescription of ACE Inhibitors or angiotensin blockers therapy, before discharge. In our practice in the absence of contraindications, it is possible to recommend prolonged beta blockers therapy with the aim of reducing the likelihood of recurrent episodes. Annual clinical followup is advisable because the natural history of apical left ventricular ballooning syndrome remains unknown. 

In conclusion, stress or Takotsubo cardiomyopathy is an increasingly recognized type of acquired cardiomyopathy occurring commonly after a recent stressful event, in peculiar emotional stress. It is typically characterized by transient myocardial systolic dysfunction that is mainly confined to the apical region of the left ventricle. The clinical presentation closely resembles that of an acute coronary syndrome, with chest pain, ischemic type ST changes, or T-wave inversion and minimal cardiac enzyme elevation. Coronary angiography and echocardiography are necessary to establish the diagnosis. The pathogenetic mechanism remains unknown although catecholamine surge plays probably a primary role. A relative deficiency of estrogen after menopause may predispose women to developing this Takotsubo syndrome. Microvascular disease and dysfunction in women may lead to myocardial ischemia in response to mental stress [15]. The most credible cause of transient and spontaneously resolving segmental myocardial stunning may be a coronary spasm, but this pathophysiological mechanism is controversial.

## Figures and Tables

**Table 1:** Mayo Clinic criteria.

(1) Transient akinesis or dyskinesis of the left ventricular apical and midventricular segments with regional wall motion abnormalities beyond a single epicardial vascular distribution	
(2) Frequent emotional or physical stress (but not absolute necessary) precedes the onset of symptoms	
(3) Absence of obstructive coronary disease or angiographic evidence of acute plaque rupture	
(4) ST segment elevation and or inversed T wave	
(5) Moderate troponin elevation	
(6) Absence of signs for pheochromocytoma, intracranial bleeding myocarditis	

Bybee et al. [3].
